# Severe Atherosclerosis and Hypercholesterolemia in Mice Lacking Both the Melanocortin Type 4 Receptor and Low Density Lipoprotein Receptor

**DOI:** 10.1371/journal.pone.0167888

**Published:** 2016-12-28

**Authors:** Vera Lede, Christin Franke, Andrej Meusel, Daniel Teupser, Albert Ricken, Joachim Thiery, Jürgen Schiller, Daniel Huster, Torsten Schöneberg, Angela Schulz

**Affiliations:** 1 Molecular Biochemistry, Rudolf-Schönheimer-Institute of Biochemistry, Medical Faculty, University of Leipzig, Leipzig, Germany; 2 Institute of Medical Physics and Biophysics, Medical Faculty, University of Leipzig, Leipzig, Germany; 3 Institute of Laboratory Medicine, Clinical Chemistry and Molecular Diagnostics, Medical Faculty, University of Leipzig, Leipzig, Germany; 4 Institute of Laboratory Medicine, Ludwig Maximilians University Munich, Munich, Germany; 5 Institute of Anatomy, Medical Faculty, University of Leipzig, Leipzig, Germany; Northeastern Ohio Medical University, UNITED STATES

## Abstract

Dysfunction of the melanocortin system can result in severe obesity accompanied with dyslipidemia and symptoms of the metabolic syndrome but the effect on vascular atherogenesis is not known. To study the impact of obesity and dyslipidemia on the cardiovascular system, we generated mice double-deficient for the melanocortin type 4 receptor (*Mc4r*^*mut*^ mice) and the LDL receptor (*Ldlr*^-/-^ mice). *Mc4r*^*mut*^ mice develop obesity due to hyperphagia. Double-mutant mice (*Mc4r*^*mut*^;*Ldlr*^-/-^) exhibited massive increases in body weight, plasma cholesterol and triacylglycerol levels and developed atherosclerosis. Atherosclerotic lesion size was affected throughout the aortic root and brachiocephalic artery not only under semisynthetic, cholesterol-containing diet but also under cholesterol-free standard chow. The *Mc4r*^mut^ mice developed a hepatic steatosis which contributes to increased plasma cholesterol levels even under cholesterol-free standard chow. Transcripts of cholesterol biosynthesis components and liver cholesterol levels did not significantly differ between wild-type and all mutant mouse strains but RNA sequencing data and biochemical measurements point to an altered bile acid elimination in *Mc4r*^*mut*^;*Ldlr*^-/-^. Therefore, the unchanged endogenous cholesterol biosynthesis together with a reduced hepatic VLDL and LDL-cholesterol clearance most likely led to increased plasma lipid levels and consequently to atherosclerosis in this animal model. Our data indicate that dysfunction of the melanocortin-regulated food intake and the resulting obesity significantly add to the proatherogenic lipoprotein profile caused by LDL receptor deficiency and, therefore, can be regarded as relevant risk factor for atherosclerosis.

## Introduction

Obesity is a global epidemic with significant morbidity and mortality affecting both adults and children. Insulin resistance, dyslipidemia and hypertension are often found in obese patients. This symptom complex is referred to as the metabolic syndrome [1] which increases the risk of diabetes and cardiovascular diseases [2]. The dyslipidemia that develops in obese humans is often characterized by elevated triacylglycerol levels, low levels of high-density lipoprotein and the appearance of small, dense low-density lipoproteins, a combination referred to as proatherogenic lipoprotein profile or "lipid triad". Although the association between obesity and dyslipidemia is well established, the mechanism by which obesity contributes to elevations of plasma lipids is not completely understood [3].

Mouse models are widely used to study obesity, the metabolic syndrome, dysfunctions of the lipid metabolism, and atherosclerosis. However, animal models that encompass all of these characteristics remain limited. Mouse strains deficient for apolipoprotein E (*apoE*) [4] and the low-density lipoprotein receptor (*Ldlr*) [5] have been broadly used as models for atherosclerosis. For example, mice deficient for the *Ldlr* (*Ldlr*^-/-^ mice) have high levels of low-density lipoprotein (LDL) and can develop massive atherosclerotic lesions depending on the diet [6]. Most importantly, *Ldlr*^-/-^ mice are models for hyperlipidemia and atherosclerosis but the effect of a metabolic syndrome cannot be addressed along with lipid disorders in this model.

The hypothalamus coordinates extra-hypothalamic regions to maintain energy homeostasis through the regulation of food intake and energy expenditure. A number of anorexigenic and orexigenic molecules in the hypothalamic nuclei participate in the control of energy homeostasis among them the proopiomelanocortin-derived melanocyte-stimulating hormones and their receptors, specifically the melanocortin receptors *Mc3r* and *Mc4r* [7]. In humans, loss-of-function mutations in *Mc4r* are associated with hyperphagia, severe early-onset obesity, increased longitudinal growth, fasting hyperinsulinemia, and increased lean mass [8–10], a phenotype that closely mirrors that seen in *Mc4r*-deficient mice [11], supporting an essential role for the melanocortin system in energy homeostasis across mammalian species [12]. Mutations in *Mc4r* are the most frequent monogenic cause of severe early-onset obesity in humans [13]. It has been demonstrated that the level of central nervous *Mc4r* activity potently and rapidly determines the balance among cellular glucose uptake, triacylglycerol synthesis, lipid deposition, and lipid mobilization in liver, muscle, and adipose tissue [14].

Several mouse strains have been established to investigate the impact of obesity on atherosclerosis. Most studies combine *apoE* or *Ldlr* deficiency with alterations of the leptin system [15–20]. There is increasing experimental evidence that leptin and the leptin receptor have specific effects on the development of atherosclerosis [17]. Although the leptin and melanocortin systems are tightly linked, we asked the question of whether obesity induced by defects in the melanocortin system has similar effects on the development of atherosclerosis. To mimic most closely the human defects in the melanocortin type-4 receptor, we bred mice carrying a partial loss-of-function missense mutation in the *Mc4r* [21] with *Ldlr*^-/-^ mice [5]. The double-mutant mice were used to study the contribution of the melanocortin system to dyslipidemia and atherosclerosis depending on the alimentary cholesterol content [5, 22]. We found that the double-mutant mice developed dyslipidemia and atherosclerosis. This mouse model shows that obesity caused by *Mc4r* deficiency can promote atherosclerosis already under cholesterol-free chow. Hepatic RNA sequencing and biochemical data suggest that hepatosteatosis and altered bile acid elimination contribute to the proatherogenic lipoprotein profile and consequently lead to the formation of atherosclerotic lesions.

## Materials and Methods

### Mouse strains and genotyping

Using the chemical random mutagenesis technique with the germline supermutagen N-ethyl-N-nitrosourea (ENU), a mouse model for mutant *Mc4r* was generated by Ingenium Pharmaceuticals AG, Martinsried, Germany. Functional *in vitro* analysis of the mouse *Mc4r* containing the mutation Ile^194^Phe revealed a partial loss of receptor function (~40-fold reduced agonist potency). At the *in vivo* level, this mutant causes the same full obese phenotype as observed in a mouse strain containing a *Mc4r* mutation (Tyr^302^Cys) with a complete loss-of-function in *in vitro* assays [21]. We used the Ile^194^Phe mouse strain (referred to as *Mc4r*^*mut*^) to closely mimic the *Mc4r* dysfunction most frequently found in humans with inhered obesity [23]. Mice were bred and maintained under specific-pathogen-free conditions at the centralised animal care facility, where lights were automatically controlled (12 h light/12 h dark). All animal experiments were conducted in accordance with the European Directive 2010/63/EU on the protection of animals used for scientific purposes and were performed with permission of the Animal Care and Use Committee (ACUC #TVV 43/07) and the Government of the State of Saxony, Germany.

The initial Ile^194^Phe C3HeB/FeJ (C3H) mouse strain [21] was crossed into the C57BL/6J (B6) background over more than 12 generations. Breeding for experiments was performed with heterozygous breeding pairs. Genotyping of the littermates was performed by polymerase chain reaction (PCR) followed by *Bsp*HI (New England Biolabs, Frankfurt, Germany) restriction analysis. The following primers and PCR conditions were used: 5’- taccctgttaaacagtacggatac-3’ (sense) and 5’- gaacatggaaatgaggcagatca-3’ (antisense) creating a *Bsp*HI-site in *Mc4r*^+/+^ sequence, conditions: 94°C 3 min; 35 cycles of 94°C 30 sec, 58°C 30 sec and 72°C 1 min. Products were digested with *Bsp*HI and fragments were separated in a 3% agarose gel.

We analyzed groups of *Mc4r*^+/+^ and *Mc4r*^*mut*^ mice of both genders on standard chow diet and a semisynthetic diet containing 0.02% cholesterol (Ssniff GmbH, Soest, Germany) [24] (S1 Table). Furthermore, *Mc4r*^*mut*^ mice were crossed onto a homozygous B6.*Ldlr*^-/-^ background (The Jackson Laboratory, Bar Harbor, Maine, stock no. 002207) to generate double-deficient mice. These (*Mc4r*^*mut*^*;Ldlr*^*-/-*^) and the respective control (*Ldlr*^-/-^) of both genders were weaned at 3 weeks of age and fed standard chow or semisynthetic diet until they were euthanized after 180 days of age. For euthanasia, mice were deeply narcotized by i.p. injection with a 100 μl mixture of 100 mg/kg body weight ketamine and 5 mg/kg body weight xylazine. Blood was collected post-euthanasia by heart puncture into syringes containing EDTA and the circulatory system was flushed with PBS (20 ml). Tissues (heart and brachiocephalic artery (BCA)) were collected from sacrificed mice and snap-frozen in Tissue-tek OCT compound (Sakura Finetek).

### Blood chemistry and histology

Enzymes, lipids and glucose were analyzed in serum and whole blood, according to the guidelines of the German Society of Clinical Chemistry and Laboratory Medicine, using a Hitachi PPEModular analyzer and an Accu-Check^®^ blood glucose measurement device (both Roche Diagnostics, Mannheim, Germany), respectively. Lipoproteins were isolated by sequential ultracentrifugation from 60 μl of plasma at densities (d) < 1.006 g/ml (very low–density lipoprotein), 1.006 ≤ d ≤ 1.063 g/ml (intermediate-density lipoprotein and low-density lipoprotein), and d > 1.063 g/ml (high-density lipoprotein) in an LE-80K ultracentrifuge (Beckman) as described [25].

Atherosclerosis quantification in the aortic root and the BCA was performed as previously described [26]. In brief, the OCT-embedded BCA was sectioned from distal to proximal at 10 μm thickness. Atherosclerotic lesions luminal to the internal elastic lamina were quantified in three equidistant oil red O-stained sections 200, 400, and 600 μm from the branching point of the BCA into the carotid and subclavian arteries. Histomorphological characterization and computerized morphometric quantification (Zeiss KS300) of the lesions was performed by two investigators, blinded to the protocol. The mean of the lesion size of the three sections was used to represent individual atherosclerosis development in the BCA. The aortic root was sectioned from proximal to distal collecting and evaluating 5 sections (each in 50 μm distance).

### Lipid extraction

Lipid extraction was conducted according to Folch et al. [27]. Frozen liver tissue (n = 5 per group, 6-month-old male) was transferred into chloroform/methanol (2:1, v/v) and vortexed for at least 1 minute. Afterwards, the tissue was sonicated on ice for 1 minute and shaken for 2 h at room temperature and 90 rpm. After shaking, 600 μl 0.9% NaCl solution was added and samples were vortexed for 1 minute. Phase separation was achieved by 10 minutes of centrifugation at 2,500 rpm. The organic phase was transferred into a new tube and vaporized by vacuum centrifugation. Complete cholesterol was determined using the enzymatic Amplex^®^ Red Cholesterol Assay Kit (Invitrogen). To determine the amount of extracted cholesterol, the pellet was solubilized in 500 μl 1× reaction buffer. A volume of 20 μl of this solution was further diluted 1:10 in 1× reaction buffer. The reaction was started by adding 50 μl of the diluted extract to 50 μl master mix which was prepared according to the assay protocol. The solution was incubated at 37°C for 30 minutes. Afterwards the samples were excited at 560 nm and fluorescence intensity was measured with an Infinite^®^ M200 TECAN Reader at 590 nm. Intensities were compared to a standard curve and the amount of cholesterol was calculated per gram of liver tissue.

### RNA sequencing of liver transcripts

Total RNA from liver (10 male mice per genotype) was extracted by using the RNeasy Micro Kit^™^ (Qiagen, Hilden, Germany) as described in the manufacturer’s instructions. The quantity of the RNA was measured using a spectrophotometer (Nanodrop ND 1000) and RNA quality of all samples was examined on the Agilent 2100 bioanalyzer using the RNA 6000 Nano Chip (Agilent Technologies, Santa Clara, CA). We only included RNA samples with a RIN value above 8.

Indexed cDNA libraries were generated using TruSeq RNA Sample Preparation Kits v2 (Illumina, San Diego, CA, USA) according to the manufacturer’s protocol, constructing libraries with an average size of 300 bp as evaluated on the Agilent 2100 bioanalyzer with DNA 1000 Chips.

Libraries were sequenced on Illumina HiScanSQ (Core Unit Sequencing, University of Leipzig), performing ten biological replicates for each genotype. 101 bp raw paired-end reads were generated on 8 flow cell lanes. Briefly, after quantification of the libraries using the Library Quantification Kit—Illumina/Universal (KAPABiosystems) according to the instructions of the manufacturer, products were used for cluster generation. Library DNA at a concentration of 10 pM was clustered using an Illumina cBot according to the PE_Amp_Lin_Block_Hybv8.0 protocol of the manufacturer. Sequencing was performed using version 3 chemistry and the version 3 flow cell according to the manufacturer’s instructions. Median cluster density was usually about 600,000 clusters per mm^2^ or 80–100 million raw clusters per lane.

After intensities call, raw reads were separated according to library indexes allowing up to one mismatch in the index sequence, but requiring that all bases have a quality score above 15 (PHRED-scale). After assigning reads to samples we used an in-house-sequencing pipeline to trim the adapters and to remove low quality reads [28]. Reads were mapped to the reference mouse genome (July 2007 NCBI37/mm9) with Ensembl v66 annotations using Tophat 2.0.6. [29, 30] which aligns reads using Bowtie2 (version 2.1.0). Mitochondrial reads and reads which did not map uniquely to a genome position were excluded. The transcription level for each gene was obtained by intersecting mapping results with gene annotations using BEDTools IntersectBed [31]. Using the DESeq software package [32], differential expression of wt and KO genes was examined. Only genes that were expressed at least in 10 animals among the groups were included for analyses. Differentially expressed genes with a nominal p-value < 0.05 were considered as statistically significant.

### Statistical analyses

For analyses of significant differences between the genotypes an unpaired two-tailed Student’s t-test was used, p-values of < 0.05 were marked *, < 0.01 **, and < 0.001 with ***. To correlate gender difference in weight gain and body length dependent on the genotypes and diets a 3-way ANOVA was used (S2 Table).

To assess the relationship between the plaque sizes (aortic root, BCA) and the total cholesterol serum levels a Pearson product-moment correlation coefficient was computed. The results were summarized in scatterplots and tested for significance.

To test for influence of different parameters on the atherosclerotic plaque size, we run a multiple regression analysis. We included the serum cholesterol levels, sex, diets, and the *Mc4r* deficiency as potential predictors and used the plaque size as response variable. The overall model including all predictors was highly significant for both, the plaque size in heart and the BCA (BCA: F_(4, 94)_ = 37.44, p-value < 2.2e^-16^ r^2^ = 0.60; heart: F_(4, 81)_ = 65.09, p-value < 2.2e^-16^, r^2^ = 0.75).

## Results

### Effects of cholesterol diet and Ldlr deficiency on increases in body weight and length in Mc4r-deficient mice

*Mc4r* deficiency led to a significant increase in body weight in male (45%) and female (50%) mice fed on standard chow and in male (30%) and female (62%) mice on a semisynthetic diet (Table 1). The body weight differences between *Mc4r*^*mut*^ and the other mouse strains were already obvious after one month after birth (S1 Fig). The difference of weight due to *Mc4r* deficiency was also found on the *Ldlr*^-/-^ background (standard chow: male 36%, female 83%; semisynthetic diet: male 37%, female 89%). Here, the weight difference between wt female mice and *Mc4r*^*mut*^;*Ldlr*^-/-^ females (standard chow: 83%; semisynthetic diet: 89%) was significantly higher as in *Mc4r*-deficient females mice (standard chow: 50%; semisynthetic diet 62%). The *Ldlr*^-/-^ alone did not significantly influence the body weight in both genders and under the different diets.

**Table 1:** Body weight and length under standard and cholesterol-containing diets. Body weights and length of the different genotypes were determined after 184 ± 3 (range 172–194) days after birth. Animals were kept on standard chow or semisynthetic diet containing 0.02% cholesterol. Data are given as means ± SD. Number of animals are given in parenthesis, and results between groups were tested for significance to the respective *Mc4r*^*+/+*^: *p < 0.05, ** p < 0.01; *** p < 0.001.

	**male (weight, gram)**		**female (weight, gram)**	
	**standard**	**semisynthetic**	**standard**	**semisynthetic**
*Mc4r*^+/+^;*Ldlr*^+/+^	31.7 ± 3.1 (10)	37.7 ± 5.3 (10)	27.4 ± 3.2 (14)	25.8 ± 2.1 (16)
*Mc4r*^*mut*^;*Ldlr*^+/+^	46.0 ± 5.1 (10)***	48.9 ± 3.5 (13)***	41.2 ± 6.6 (11)***	41.9 ± 3.8 (11)***
*Mc4r*^+/+^;*Ldlr*^-/-^	32.2 ± 2.6 (17)	37.3 ± 4.4 (16)	23.3 ± 1.4 (15)	25.3 ± 2.2 (18)
*Mc4r*^*mut*^;*Ldlr*^-/-^	43.9 ± 8.1 (15)***	51.3 ± 3.8 (16)***	42.7 ± 5.6 (15)***	47.9 ± 6.9 (14)***
	**male (length, cm)**		**female (length, cm)**	
	**standard**	**semisynthetic**	**standard**	**semisynthetic**
*Mc4r*^+/+^;*Ldlr*^+/+^	9.6 ± 0.6 (10)	10.0 ± 0.3 (14)	9.6 ± 0.6 (12)	9.4 ± 0.3 (13)
*Mc4r*^*mut*^;*Ldlr*^+/+^	10.2 ± 0.3 (10)**	10.4 ± 0.3 (9)*	10.2 ± 0.3 (11)***	10.2 ± 0.2 (11)***
*Mc4r*^+/+^;*Ldlr*^-/-^	9.7 ± 0.4 (17)	9.7 ± 0.3 (14)	9.1 ± 0.3 (13)	9.1 ± 0.4 (18)
*Mc4r*^*mut*^;*Ldlr*^-/-^	10.2 ± 0.3 (15)**	10.4 ± 0.3 (16)***	9.8 ± 0.4 (14)***	10.0 ± 0.3 (14)***

In our mouse strain male and female *Mc4r*^*mut*^ animals showed a slightly increased body length of 6% compared to wild-type controls (Table 1). The difference of length between wt and *Mc4r*^*mut*^ was also found on the semisynthetic diet in male (4%) and female (8%) mice. The difference of length due to *Mc4r* deficiency was also found on the *Ldlr*^-/-^ background (standard chow: male 5%, female 8%; cholesterol diet: male 7%, female 10%).

The gender differences in weight gain and body length depending on the genotypes and diet were tested using a 3-way ANOVA. It revealed a significant interaction between genotype and sex (F_3, 182_ = 5.6579, p < 0.001) which was mainly driven by the differences of females and males on the *Mc4r*^*mut*^ background. In all cases the differences of body weight and length in female *Mc4r*^*mut*^ were higher than in male *Mc4r*^*mut*^ (S2 Table, S2 and S3 Figs).

In summary, *Mc4r*-deficient mice showed the expected obese phenotype with gender-specific characteristics. The combination of *Mc4r* and *Ldlr* deficiencies had no major impact on the development of obesity in the *Mc4r*^*mut*^ mouse strains.

### Dyslipidemia in mice lacking both receptors Mc4r and Ldlr

**Fig 1:**
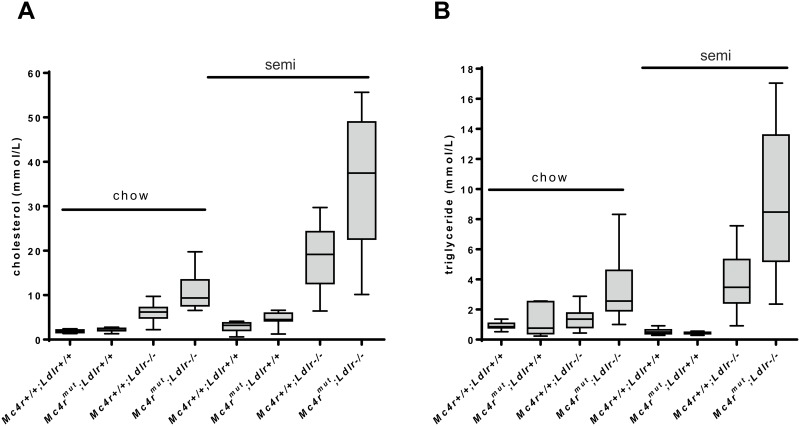
Severe hypercholesterolemia and hypertriglyceridemia in mice lacking both MC4 and LDL receptors. Serum cholesterol (**A**) and serum triglycerides (**B**) were determined in the indicated mouse strains (n = 10–14 male mice). Female mice show essentially similar results (see Table 2). The statistic evaluation is given in Table 2.

Next, we addressed the question whether *Mc4r* deficiency contributes to increases in plasma lipids in *Ldlr*^-/-^ mice and, therefore, can be considered as a risk factor of atherosclerosis. As shown in Fig 1 and Table 2, *Mc4r* deficiency alone caused small and moderate increases in plasma cholesterol levels under standard chow and semisynthetic cholesterol-containing diet, respectively, in both genders. The increase in cholesterol was most pronounced in the LDL fraction (Table 2). The triglyceride levels remained normal in *Mc4r*^*mut*^ on both diets. Essentially similar results were found in a *Mc4r* gene-deletion mouse strain but the cholesterol content of the diet was not stated [33].

**Table 2:** Plasma lipids and lipoproteins and liver cholesterol on standard chow and semisynthetic diets. Plasma lipids and liver cholesterol content of the different genotypes were determined after 184 ± 3 days after birth. Animals were kept on standard chow and semisynthetic diet. Data are given as means ± SD and tested for significance to the respective *Mc4r*^+/+^: *p < 0.05, ** p < 0.01; *** p < 0.001. The number of animals per group was between 10 and 14 (plasma lipids) and 5 (liver cholesterol). n.d., not determined.

	Male				Female			
	Mc4r^+/+^;Ldlr^+/+^	Mc4r^mut^;Ldlr^+/+^	Mc4r^+/+^;Ldlr^-/-^	Mc4r^mut^;Ldlr^-/-^	Mc4r^+/+^;Ldlr^+/+^	Mc4r^mut^;Ldlr^+/+^	Mc4r^+/+^;Ldlr^-/-^	Mc4r^mut^;Ldlr^-/-^
**plasma cholesterol (mmol/L)**								
standard chow	1.90 ± 0.33	2.13 ± 0.67*	5.71 ± 2.01	10.74 ± 3.81***	1.49 ± 0.27	2.04 ± 0.39**	5.11 ± 1.04	7.90 ± 2.39***
semisynthetic diet	2.87 ± 1.06	4.64 ± 1.34***	20.17 ± 11.30	35.54 ± 14.05**	1.80 ± 0.42	2.44 ± 0.51**	15.72 ± 6.47	30.36 ± 12.06***
**VLDL cholesterol (mmol/L)**								
standard chow	0.16 ± 0.06	0.11 ± 0.09	0.53 ± 0.25	1.92 ± 1.46**	0.11 ± 0.07	0.09 ± 0.06	0.53 ± 0.58	1.48 ± 0.92**
semisynthetic diet	0.11 ± 0.09	0.17 ± 0.11	4.34 ± 3.23	9.83 ± 4.32***	0.08 ± 0.06	0.08 ± 0.07	4.69 ± 3.25	7.62 ± 4.60
**LDL cholesterol (mmol/L)**								
standard chow	0.65 ± 0.14	0.91 ± 0.34***	3.66 ± 1.65	8.13 ± 3.00***	0.58 ± 0.13	0.83 ± 0.19**	3.67 ± 0.85	6.11 ± 2.12**
semisynthetic diet	1.30 ± 0.58	2.08 ± 0.77**	14.11 ± 5.86	23.11 ± 5.89***	0.74 ± 0.40	0.98 ± 0.30	10.40 ± 4.21	18.53 ± 6.91**
**HDL cholesterol (mmol/L)**								
standard chow	1.18 ± 0.23	1.21 ± 0.35	1.43 ± 0.40	1.40 ± 0.48	0.90 ± 0.16	1.16 ± 0.12***	1.14 ± 0.37	1.40 ± 1.46
semisynthetic diet	1.65 ± 0.59	2.75 ± 1.35*	1.67 ± 0.50	2.00 ± 0.60	1.06 ± 0.22	1.38 ± 0.26**	1.17 ± 0.38	1.57 ± 0.32**
**plasma triglyceride (mmol/L)**								
standard chow	0.92 ± 0.23	1.11 ± 0.88	1.38 ± 0.60	3.44 ± 2.15**	0.58 ± 0.16	0.54 ± 0.17	1.14 ± 0.61	2.33 ± 1.29**
semisynthetic diet	0.53 ± 0.19	0.43 ± 0.08	4.72 ± 4.44	9.08 ± 4.61**	0.47 ± 0.10	0.37 ± 0.11*	2.74 ± 1.46	6.37 ± 3.04***
**VLDL triglyceride (mmol/L)**								
standard chow	0.63 ± 0.29	0.27 ± 0.25*	0.65 ± 0.32	2.63 ± 2.22**	0.28 ± 0.16	0.17 ± 0.10	0.45 ± 0.36	1.58 ± 1.44**
semisynthetic diet	0.22 ± 0.18	0.15 ± 0.09	3.22 ± 1.98	5.51 ± 1.91**	0.17 ± 0.08	0.15 ± 0.07	1.72 ± 1.13	3.49 ± 2.21*
**LDL triglyceride (mmol/L)**								
standard chow	0.32 ± 0.09	0.31 ± 0.16	0.61 ± 0.23	1.13 ± 0.53**	0.28 ± 0.14	0.22 ± 0.05	0.50 ± 0.16	0.79 ± 0.34**
semisynthetic diet	0.40 ± 0.25	0.34 ± 0.22	1.23 ± 0.57	2.13 ± 0.82***	0.37 ± 0.15	0.28 ± 0.10	0.76 ± 0.31	1.36 ± 0.47**
**HDL triglyceride (mmol/L)**								
standard chow	0.11± 0.06	0.19 ± 0.18	0.13 ± 0.07	0.29 ± 0.20*	0.18 ± 0.15	0.13 ± 0.05	0.17 ± 0.30	0.21 ± 0.17
semisynthetic diet	0.29 ± 0.26	0.33 ± 0.35	0.32 ± 0.33	0.66 ± 0.46*	0.31 ± 0.20	0.19 ± 0.15	0.19 ± 0.13	0.39 ± 0.21**
**liver cholesterol (mg/g liver)**								
standard chow	0.64 ± 0.15	0.88 ± 0.19	0.67 ± 0.16	0.72 ± 0.34	n.d.	n.d.	n.d.	n.d.
semisynthetic diet	0.85 ± 0.16	0.93 ± 0.24	0.90 ± 0.48	0.61 ± 0.34				

As shown in Fig 1 and Table 2, the combination of both receptor defects caused massive increases in plasma cholesterol and triglyceride levels. Importantly, this effect was already seen under standard chow which did not contain detectable amounts of cholesterol. The cholesterol and triglyceride levels were higher in male compared to female mice. Significant increases in lipids were found in all lipoprotein fractions (Table 2).

### MC4R-deficient mice develop hepatic steatosis independent of plasma cholesterol levels

Because cholesterol and triglycerides are mainly produced in the liver, we focused our analysis on hepatic functional parameters. As shown in Table 3, *Mc4r*^*mut*^ mice present significantly elevated activities of ASAT, ALAT, GLDH, and CHE. Interestingly, enzyme activities were further increased by semisynthetic diet but not by *Ldlr* deficiency (see Table 3). Liver morphology and histology revealed a massive hepatic steatosis without histological signs of inflammation (see accompanied paper Lede et al.) as already described for *Mc4r*^*mut*^ [34, 35]. This non-alcoholic fatty liver disease (NAFLD) may slowly progress into non-alcoholic steatohepatitis (NASH), which is characterized by excessive liver inflammation. It has been shown that cholesterol is an important risk factor for the progression to hepatic inflammation in diet-induced NASH [36].

**Table 3:** Blood glucose, serum urea levels, and liver enzymes on standard chow and semisynthetic diets. Enzyme activities of the different genotypes were determined after 184 ± 3 days after birth. Animals were kept on standard chow and semisynthetic diet. Data are given as means ± SD and tested for significance to the respective *Mc4r*^+/+^: * p < 0.05, ** p < 0.01; *** p < 0.001. The number of animals/group is between 10 and 14.

	Male				Female			
	Mc4r^+/+^;Ldlr^+/+^	Mc4r^mut^;Ldlr^+/+^	Mc4r^+/+^;Ldlr^-/-^	Mc4r^mut^;Ldlr^-/-^	Mc4r^+/+^;Ldlr^+/+^	Mc4r^mut^;Ldlr^+/+^	Mc4r^+/+^;Ldlr^-/-^	Mc4r^mut^;Ldlr^-/-^
**glucose (mmol/L)**								
standard chow	7.2 ± 0.5	7.1 ± 1.2	8.0 ± 2.1	8.4 ± 2.9	6.3 ± 0.6	6.7 ± 1.3	6.8 ± 1.7	7.8 ± 1.3
semisynthetic diet	7.1 ± 1.2	7.1 ± 1.0	7.3 ± 0.9	7.0 ± 0.6	6.5 ± 0.6	7.0 ± 0.9	6.9 ± 0.6	7.2 ± 1.1
**urea (mmol/L)**								
standard chow	9.2 ± 2.2	8.9 ± 2.6	8.6 ± 2.3	8.0 ± 2.4	9.6 ± 1.8	9.3 ± 1.9	7.5 ± 2.3	9.7 ± 2.7*
semisynthetic diet	8.9 ± 1.7	8.3 ± 1.3	7.5 ± 1.8	7.4 ± 1.4	8.3 ± 1.3	9.1 ± 2.0	8.1 ± 2.1	9.5 ± 2.9
**ALAT μkat/L**								
standard chow	0.42 ± 0.09	0.92 ± 0.54**	0.62 ± 0.45	2.17 ± 1.92*	0.54 ± 0.12	1.16 ± 0.66*	0.54 ± 0.25	0.86 ± 0.63
semisynthetic diet	1.50 ± 1.44	5.28 ± 3.05**	1.60 ± 1.22	6.07 ± 2.98***	0.67 ± 0.32	3.04 ± 1.79***	0.78 ± 0.36	3.56 ± 1.55***
**ASAT μkat/L**								
standard chow	1.65 ± 0.71	1.87 ± 0.58	2.37 ± 1.04	3.73 ± 3.36	2.95 ± 1.66	2.41 ± 1.04	2.16 ± 1.26	2.75 ± 1.35
semisynthetic diet	3.38 ± 2.50	6.75 ± 4.16*	3.66 ± 1.82	7.09 ± 3.47*	3.65 ± 2.28	6.55 ± 2.95*	2.85 ± 1.08	6.37 ± 1.81***
**CHE μkat/L**								
standard chow	78.8 ± 7.66	103.9 ± 31.7***	84.2 ± 15.1	124.4 ± 20.0***	113.5 ± 11.0	135.0 ± 17.7**	114.3 ± 12.4	119.2 ± 21.7
semisynthetic diet	105.7 ± 22.8	165.2 ± 26.7***	124.4 ± 26.0	128.8 ± 28.7***	133.8 ± 18.1	171.3 ± 20.1***	145.2 ± 19.1	190.8 ± 38.0***
**GLDH μkat/L**								
standard chow	0.13 ± 0.06	0.30 ± 0.24*	0.22 ± 0.19	1.30 ± 1.39*	0.13 ± 0.06	0.69 ± 0.58*	0.12 ± 0.04	0.40 ± 0.05*
semisynthetic diet	0.54 ± 0.35	2.07 ± 0.97***	0.76 ± 0.72	2.52 ± 1.06***	0.27 ± 0.18	1.05 ± 0.75**	0.31 ± 0.24	1.92 ± 1.14***

*Mc4r*^*mut*^ mice develop a late-onset hyperglycemia [11] and normal basal blood glucose levels but increased insulin levels and hepatic insulin resistance have been reported [37, 38]. We found no significant effects on basal blood glucose levels in all genotypes under the two diets (Table 3). Interestingly, there were no significant differences in liver cholesterol between the mouse strains and both diets (Table 2) and no significant correlation between the liver cholesterol and the serum cholesterol concentrations.

### Severe atherosclerosis in mice lacking both Mc4r and Ldlr

**Fig 2:**
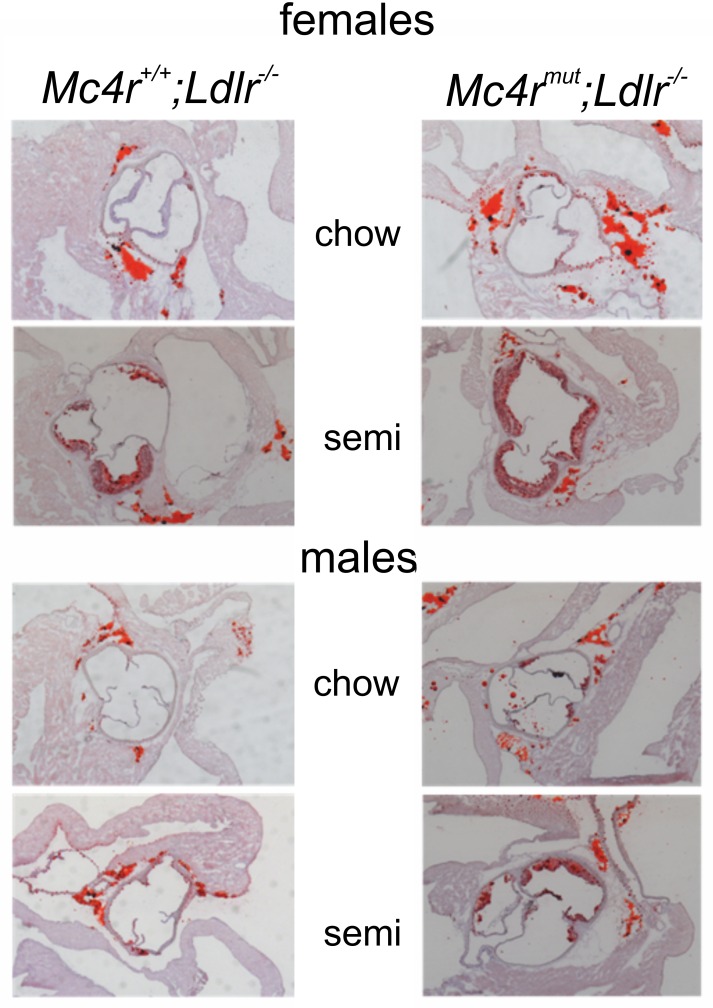
Atherosclerosis in the aortic root in mice lacking Ldlr and Mc4r;Ldlr. Representative sections of the aortic root prepared from the different mouse strains. Depicted are sections of the aortic root of female and male *Mc4r*^*mut*^ and *Mc4r*^*mut*^;*Ldlr*^-/-^ mouse strains fed a chow and semisynthetic diet (0.02% cholesterol).

**Fig 3:**
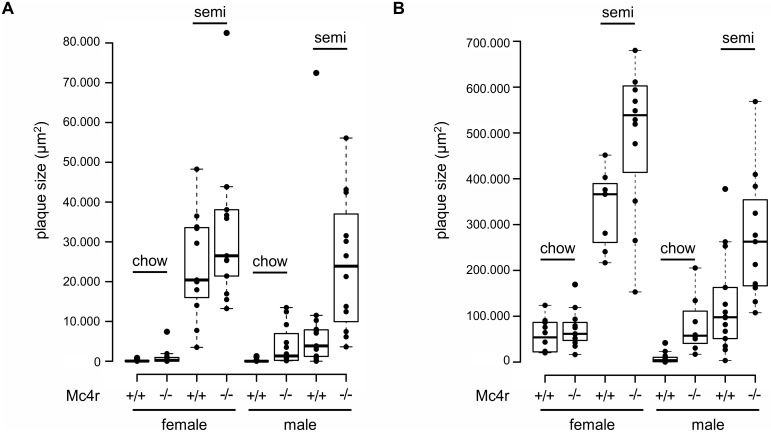
Quantification of atherosclerotic lesions at the aortic root and brachiocephalic artery (BCA). Male and female mice (wt: +/+, *Mc4r* deficiency: mut), all on an *Ldlr*-deficient background, were fed with standard chow and semisynthetic diet. Atherosclerosis quantification in the BCA (A) and the aortic root (B) was performed after 6 months as previously described (14). Data of the indicated genotypes are given as a scatter and box plot showing the median (bold line) and the lower and the upper quartile (as box) (7–15 animals per group; from left to right: n = 11, 12, 11, 13, 13, 12, 15, 12, 10, 11, 7, 12, 13, 8, 14, 11).

We analyzed atherosclerotic plaque formation in the aortic root and the BCA as pathomorphological readout for atherosclerosis in our mouse strains. No atheromata were found in mice with an *Ldlr*^+/+^ genotype regardless of the diet. Although significantly elevated cholesterol levels (up to 4 mmol/L in *Mc4r*^*mut*^ on the semisynthetic diet) and obesity *Mc4r*^*mut*^ mice did not develop atherosclerotic plaques. However, the combination of both receptor defects significantly increased atheroma formation compared to the atherosclerosis found in *Ldlr*^-/-^ mice (Figs 2, 3 and S5). Strikingly, atherosclerotic plaque formation was already found in *Mc4r*^*mut*^;*Ldlr*^-/-^ on standard chow as quantified in histological sections stained with Sudan red (Fig 3). Staining of aortic root sections with an anti-CD68 antibody, a marker expressed in macrophages, revealed macrophage-positive plaque formation in female *Mc4r*^*+/+*^;*Ldlr*^-/-^ and *Mc4r*^*mut*^;*Ldlr*^-/-^ mice of both gender (S6 Fig). Quantitative PCR analysis of the macrophage chemoattractant protein MCP1 (CCL2) showed detectable MCP1 expression in male mice on an *Ldlr*^-/-^ background, whereas MCP1 was detectable in all female groups (S7 Fig) with significant increases in atherosclerotic animals. TNF-α expression was found in aortic roots of semisynthetic fed mice (S7 Fig).

To examine the relationship between plasma cholesterol levels and the atherosclerotic plaque size, we run a multiple regression analysis. Thus, we included the serum cholesterol levels, sex, diets and the Mc4r deficiency as potential predictors and used the plaque size as response variable. The overall model including all predictors was highly significant for both, the plaque size in the aortic root and BCA (BCA: F(4, 94) = 37.44, p-value < 2.2e-16 r2 = 0.60; heart: F(4, 81) = 65.09, p-value < 2.2e-16, r2 = 0.75). Only cholesterol levels, diet and sex had a significant impact whereas the Mc4r deficiency did not reach significance (see S3 Table).

### Transcriptional changes in cholesterol and bile acid syntheses

As stated above, no differences in liver cholesterol levels were observed between the mouse strains. To further substantiate this finding, we analyzed the transcript levels of components relevant for cholesterol and bile acid biosynthesis by RNA-Seq technology. Approximately 30 million reads per animal (10 animals per genotype) were analyzed. Interestingly, the number of *Ldlr* transcripts was significantly increased in all *Ldlr*^-/-^ strains (Table 4). Detailed analysis revealed that all transcripts mapped to exons 1–4 of the *Ldlr* whereas *Ldlr* transcripts of wt mice were almost equally distributed over all exons. Comparison of transcripts mapping only to exon 1–4 revealed an approximately 10-fold increase in *Ldlr*^-/-^ strains. The *Ldlr*^-/-^ mouse strain was generated by insertion of a neomycin resistance cassette into exon 4 [5]. The targeted allele would encode a truncated non-functional protein that will not bind LDL, and lacks a membrane spanning segment. Immunoblot analysis of liver membranes detected a truncated protein in homozygous mutant animals [5]. This nicely fits with the RNA-Seq data and upregulation can be interpreted as a compensatory regulation because of the lack of *Ldlr* function.

**Table 4:** Transcriptional changes of selected components of the cholesterol and bile acid biosyntheses. RNA sequencing was performed with RNA from livers of 10 animals of each genotype. Means of the expression ratio (KO/WT) for each gene are listed. For p-value calculations an unpaired two-tailed Student’s t-test was used.

	WT vs Mc4r^mut^		WT vs Ldlr^-/-^		WT vs Mc4r^mut^;Ldlr^-/-^		Ldlr^-/-^ vs Mc4r^mut^;Ldlr^-/-^	
transcript	fold change WT—KO	p-value	fold change WT—KO	p-value	fold change WT—KO	p-value	fold change WT—KO	p-value
Ldlr	1.33	0.12	**3.31**	0.001	**3.49**	0.001	1.05	0.609
Srebf1	1.37	0.43	0.86	0.428	1.1	0.634	1.27	0.092
Srebf2	0.37	0.068	0.31	0.066	**0.3**	0.034	0.94	0.44
Scd1	**2.41**	0.005	1.39	0.377	**2.19**	0.024	1.56	0.053
Fasn	2.29	0.195	0.96	0.421	1.74	0.256	**1.8**	0.025
Ppara	1.16	0.576	0.96	0.253	1.14	0.971	1.18	0.197
Pparg	**2.35**	0.001	0.92	0.784	**2.13**	0.001	**2.3**	0.001
**Cholesterol biosynthesis**								
Cyp51	0.87	0.085	0.92	0.464	0.73	0.051	0.79	0.133
Dhcr24	**1.58**	0.003	1.05	0.49	1.28	0.112	1.21	0.275
Dhcr7	1.07	0.871	0.9	0.807	0.87	0.437	0.96	0.475
Hmgcr	1.1	0.822	0.87	0.799	0.88	0.956	1.01	1
Hmgcs2	1.32	0.064	1.03	0.5	1.3	0.34	1.25	0.122
Fdft1	1.16	0.666	0.95	0.887	0.87	0.621	0.91	0.396
Hsd17b7	0.89	0.116	**0.78**	0.026	**0.75**	0.028	0.96	0.766
Lss	1.05	0.532	0.91	0.649	0.91	0.557	1	0.771
Mvk	0.85	0.274	0.72	0.06	**0.55**	0.012	0.77	0.105
Nsdhl	0.97	0.318	0.92	0.509	0.85	0.19	0.91	0.433
Sc4mol	0.78	0.052	0.87	0.461	**0.66**	0.025	0.76	0.053
Sqle	0.94	0.334	1.09	0.257	0.8	0.239	**0.73**	0.027
Tm7sf2	1.07	1	1.01	0.796	0.94	0.546	0.93	0.705
**Bile Acid biosynthesis**								
Acot8	**1.36**	0.029	1.05	0.655	1.14	0.484	1.08	0.731
Acox2	0.95	0.696	0.98	0.826	0.86	0.342	0.87	0.387
Akr1d1	0.94	0.327	1.23	0.343	1.19	0.238	0.97	0.685
Amacr	0.94	0.898	1.01	0.963	0.87	0.403	0.86	0.323
Baat	0.86	0.187	0.96	0.618	0.77	0.11	0.8	0.187
Cyp27a1	0.95	0.97	0.88	0.506	0.71	0.061	0.8	0.115
Cyp39a1	0.65	0.017	1.04	0.858	0.88	0.469	0.84	0.325
Cyp46a1	4.02	0.019	2.19	0.084	4.45	0.008	2.02	0.058
Cyp7a1	0.69	0.436	0.68	0.615	0.62	0.602	0.91	0.744
Cyp7b1	0.35	0.001	0.53	0.008	0.21	0.001	0.4	0.004
Cyp8b1	1.32	0.03	0.73	0.009	0.94	0.941	1.28	0.038
Hsd17b4	1.21	0.169	0.9	0.205	1.1	0.579	1.22	0.091
Hsd3b7	0.97	0.576	0.92	0.243	0.8	0.155	0.87	0.608
Scp2	1.03	0.797	0.93	0.551	0.87	0.591	0.93	0.849
Scp2-ps2	1.39	0.776	0.73	0.066	1.17	0.398	1.6	0.289
Slco1a1	**0.48**	0.001	0.81	0.053	**0.26**	0.001	**0.32**	0.004
Slc27a5	0.93	0.77	1.03	0.744	0.9	0.554	0.87	0.33

Several studies on mice with hepatic steatosis have shown that several transcription factors and components of the triacylglycerol metabolism such as PPARγ (Pparg), fatty acid synthetase (Fasn), SREBP-1c (Srebf1) and stearoyl-coenzyme A desaturase 1 (Scd1) are increasingly expressed in the liver. We confirmed some of them in our mouse strains where *Mc4r*^*mut*^ and *Mc4r*^*mut*^*;Ldlr*^-/-^ showed significantly higher mRNA levels for PPARγ and stearoyl-coenzyme A desaturase 1 compared to wild-type animals (Table 4). The transcripts of fatty acid synthetase and SREBP-1c were higher in those mouse strains but values reached no significance. The detailed analysis of the complete liver transcriptome data under the different diets and changes in liver lipid compositions is presented in the accompanied manuscript Lede et. al.,).

In congruence with unchanged hepatic cholesterol we found no evidence for significant changes in components of the cholesterol biosynthesis (Table 4). Only the mevalonate kinase (Mvk), 3-ketosteroid reductase (Hsd17b7) and sterol-C4-methyl oxidase (Sc4mol) in double KO showed significant reduction in transcript expression levels. However, the transcript level of the rate-limiting enzyme of the cholesterol biosynthesis, the HMG-CoA reductase (Hmgcr), remained unchanged.

The synthesis of primary bile acids from cholesterol occurs via two pathways: the classic neutral pathway involving cholesterol 7-alpha-hydroxylase (Cyp7a1), and the acidic pathway involving a distinct microsomal oxysterol 7-alpha-hydroxylase (Cyp7b1). Transcriptome analysis revealed a significant reduction of Cyp7b1 in all mutant mice compared to wt mice (Table 4). Also, Cyp7a1 transcript was in general lower in all mutant mice livers but did not reach significance. Interestingly, the bile acid transporter Slco1a1 was significantly downregulated in all *Mc4r*^*mut*^ strains (Table 4). To evaluate if changes in transcript levels of these enzymes result in differences of bile acid production, we analysed the bile acid content of the liver and of bile collected from the gall bladder. We found reduced (but not significant) bile acid concentrations in the liver of the *Mc4r*^*mut*^*;Ldlr*^-/-^ compared to WT animals (S4A Fig). As shown in S4B Fig, comparison between *Mc4r*^*mut*^ *and Mc4r*^+/+^ groups, showed no significant differences in bile acids in the collected bile (p = 0.38, WT vs. *Mc4r*^*mut*^; p = 0.28, *Ldlr*^-/-^ vs. *Mc4r*^*mut*^*;Ldlr*^-/-^). However, significant lower bile acid levels where found in the *Ldlr*^-/-^ and *Mc4r*^*mut*^;*Ldlr*^-/-^ strains when compared to WT animals (p = 0.037; p = 0.012).

## Discussion

**Fig 4:**
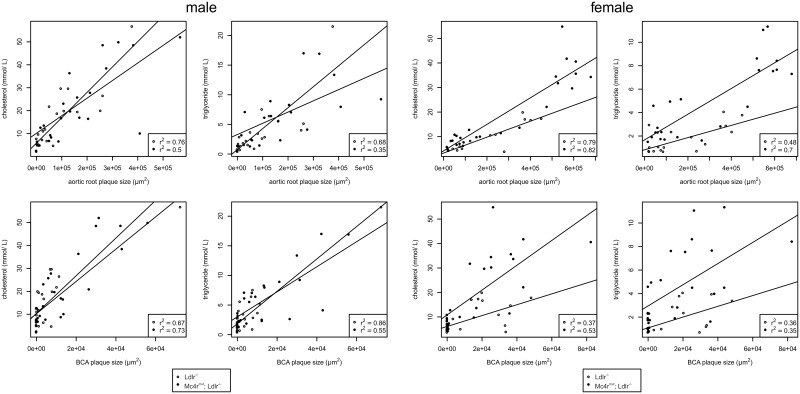
Correlation between plasma cholesterol levels and plaque sizes of atherosclerotic lesions at the aortic root and brachiocephalic artery (BCA). Correlations were performed with the data of Table 2 and Fig 3. To illustrate the results of the multiple regression analysis (see Material and Methods), we plotted the cholesterol levels against the plaque size for males and females. Furthermore, we added a linear regression line computed separately for *Ldlr*^*-/-*^ with and without *Mc4r*^*mut*^ (regression coefficients are given in the figure, the respective p-values can be found in S2 Table).

Obesity and the metabolic syndrome are well-established cofactors for the development of atherosclerosis but usually obese mice strains present vascular atheromata only by additional genetic defects in atherogenic factors (*Ldlr*, *apoE*) and cholesterol-containing diets [15–20]. Similarly, *Mc4r* deficiency leads to obesity but had only small effects on plasma lipid levels (Fig 1, Table 2) and no atherosclerotic plaques were found on standard chow and semisynthetic diet. However, combination with *Ldlr* deficiency significantly increased both, plasma triglycerides and cholesterol levels, and double-deficient male mice develop atherosclerosis already on cholesterol-free standard chow (Fig 3). This is indeed striking since atherosclerotic plaques are usually absent in atherogenic transgenic mouse strains when fed diets without cholesterol. Since the development of atherosclerosis correlated with cholesterol levels (Fig 4, S4 Table) one can consider the cause of atherosclerosis found already under cholesterol-free diet as an additive effect of both gene defects rather than as an *Mc4r*-specific, lipid-unrelated mechanism. This is supported by the multiple regression analysis indicating no significant impact of *Mc4r* deficiency itself on plaque size (S3 Table).

It is important to note that the elevated plasma cholesterol levels must have come from endogenous synthesis in mice on standard chow. However, the cholesterol content of the liver remained unchanged regardless the diet and genotype (see Table 2). While it is clear that the *Mc4r*^*mut*^;*Ldlr*^-/-^ mice have much higher triacylglycerol levels (Table 2), and because there is no high affinity clearance of apoB lipoproteins, it is either more VLDL secretion or decreased lipolysis of VLDL triacylglycerol and cholesterol causing the overall increase in lipid levels. Because decreased lipolysis probably would not increase LDL cholesterol dramatically this is most likely an obesity-driven increase in VLDL triacylglycerol and cholesterol secretion leading to the rest of the lipid changes in blood.

In a previous genetic approach the combination of leptin (*ob/ob*) and *Ldlr* deficiency lead to a very similar phenotype [16]. It was speculated that the hypertriglyceridemia and hypercholesterolemia in the double mutant mice were caused by distinct mechanisms and point to the possibility that leptin might have some impact on plasma cholesterol metabolism, possibly through an *Ldlr*-independent pathway. We can rule out this hypothesis in our double mutant mice because, in contrast to *ob/ob* mice, *Mc4r*-deficient mice exhibit very high serum leptin levels [39]. It is very likely that the hyperphagia-induced obesity in *ob/ob* and in our *Mc4r*^*mut*^ causes the hypertriglyceridemia. As the obese *ob/ob* mice, *Mc4r*-deficient mice and rodents with a pharmacological blockade of central nervous MC receptors develop an NAFLD probably due to a hepatic overproduction of triglycerides [14, 16, 34, 38, 40]. Increased expression levels of factors relevant for hepatic triglyceride metabolism such as SREBP-1c and Fasn were found in these and our studies (Table 4). *Mc4r*-deficient mice have hyperinsulinemia [34, 41] and hyperinsulinemia stimulates expression of lipogenic genes such as SREBP-1c and PPARγ in liver [42–44]. It has been shown in wt and *ob/ob* mice that insulin increases hepatic VLDL secretion. This all is supportive for a scenario in which the high food intake in *Mc4r*^*mut*^ mice increases hepatic triglyceride biosynthesis and VLDL secretion resulting in proatherogenic plasma lipoprotein profile. This is further supported by the fact that changes in lipid metabolism of *Mc4r*^*mut*^ mice mostly explain atherosclerosis. Although specific effects of *Mc4r* deficiency on atherosclerosis, especially in male mice on *Ldlr*^-/-^ background (Fig 3), cannot be completely excluded albeit statistical correlations indicated no significant influence of the genotype.

Atherosclerosis was seen already under cholesterol-free diet and the question remains what is the pathomechanism underlying the hypercholesterolemia specifically in the case of the cholesterol-free diet? We and others [16] did not find elevated cholesterol levels in the liver and RNA-sequencing data did not provide evidence for increased mRNA levels of proteins involved in cholesterol biosynthesis in *Mc4r*^*mut*^;*Ldlr*^-/-^(Table 4). Therefore, hypercholesterolemia in *Mc4r*^*mut*^;*Ldlr*^-/-^ on standard chow is not mainly caused by a hepatic overproduction but rather a result of the loss of hepatic VLDL and LDL-cholesterol clearance due to *Ldlr* deficiency. However, the combination of both, *Ldlr* and *Mc4r* deficiencies, showed additive effects on plasma cholesterol levels indicating that NALFD found in *Mc4r*^*mut*^ additionally contribute hypercholesterolemia. Reduced elimination of cholesterol via bile acid biosynthesis and excretion may be one factor. In fact, the most prominent down-regulated components in the liver of obese *ob/ob* mice [45] and of our *Mc4r*^*mut*^ strains (Table 4) were Cyp7b1 and Slco1a1. A similar observation was reported from Nagoya-Shibata-Yasuda (NSY) mice which develop type 2 diabetes and hepatic steatosis [46]. Transient overexpression of Cyp7b1 and Slco1a1 reduced weight gain in obese mice and normalized plasma glucose levels [45]. It remains speculative whether a reduced Cyp7b1 and Slco1a1 expression contributed to the additive effects on plasma cholesterol levels in double KO mice since Cyp7b1 contributes to only ~5% of total bile production and Slco1a1 is not the only bile acid transporter in liver. However, measurement of bile acid concentrations in the gall bladder of the mouse groups support the results from RNA sequencing, pointing to reduced bile acid elimination in double KO mice (S4 Fig).

In sum, most obese genetic mouse models do not develop an atherosclerosis phenotype without additional genetic defects combined with cholesterol in their diets. The *Mc4r*^*mut*^*;Ldlr*^*-/-*^ mouse strain is a rare genetic model presenting atherosclerosis already under cholesterol-free diet. The combination of the hepatic steatosis caused by *Mc4r* deficiency and a reduced hepatic VLDL and LDL-cholesterol clearance due to *Ldlr* deficiency directly correlate with plasma cholesterol levels and, therefore, with the development of atherosclerosis.

## Supporting Information

S1 Methods Additional methods. (DOCX)

S1 Table Composition of the diets used. (DOCX)

S2 Table Anova analysis. (DOCX)

S3 Table Results of the multiple regression analysis for plaque size measured in BCA and heart. (DOCX)

S4 Table Results of spearman correlation between serum cholesterol levels and the plaque size in BCA and heart, respectively. (DOCX)

S1 Fig Body weight development. (DOCX)

S2 Fig Body weight of all groups. (DOCX)

S3 Fig Body length of all groups. (DOCX)

S4 Fig Measurement of bile acids. (DOCX)

S5 Fig Atherosclerosis in mice lacking both receptors *Mc4r* and *Ldlr*. (DOCX)

S6 Fig Immunhistochemistry of hearts of *Mc4r* wildtype and mutant mice. (DOCX)

S7 Fig Gene expression of inflammatory markers in aortae. (DOCX)

## Abbreviations

apoE: apolipoprotein E
BCA: brachiocephalic artery
KO: knockout
Ldlr: low density lipoprotein receptor
Mc4r: melanocortin type 4 receptor
*Mc4r^mut^*: melanocortin type 4 receptor with missense mutation Ile^194^Phe
wt: wild-type
