# Supplementary material for: Severe Atherosclerosis and Hypercholesterolemia in Mice Lacking Both the Melanocortin Type 4 Receptor and Low Density Lipoprotein Receptor
Source: PLoS One. 2016 Dec 28;11(12):e0167888. doi: 10.1371/journal.pone.0167888 (PMC5193345; doi:10.1371/journal.pone.0167888)
Supplement: S1 Methods — (DOCX) [file pone.0167888.s001.docx]

**Supplementary Information**

**Severe atherosclerosis and hypercholesterolemia in mice lacking both the melanocortin type 4 receptor and low density lipoprotein receptor**

**Vera Lede^1^** **^¶^, Christin Franke^1¶^, Andrej Meusel^1,2^, Daniel Teupser^3,4^, Albert Ricken^5^, Joachim Thiery^3^, Jürgen Schiller^2^, Daniel Huster^2^, Torsten Schöneberg^1*^, and Angela Schulz^1*^**

^1^Molecular Biochemistry, Rudolf-Schönheimer-Institute of Biochemistry, Medical Faculty, University of Leipzig, Leipzig, Germany

^2^Institute of Medical Physics and Biophysics, Medical Faculty, University of Leipzig, Leipzig, Germany

^3^Institute of Laboratory Medicine, Clinical Chemistry and Molecular Diagnostics, Medical Faculty, University of Leipzig, Leipzig, Germany

^4^Institute of Laboratory Medicine, Ludwig Maximilians University Munich, Munich, Germany

^5^Institute of Anatomy, Medical Faculty, University of Leipzig, Leipzig, Germany

Running head: Atherosclerosis and *Mc4r* deficiency

*Corresponding authors

E-mail: [schoberg@medizin.uni-leipzig.de](mailto:schoberg@medizin.uni-leipzig.de) (TS); [angela.schulz@medizin.uni-leipzig.de](mailto:angela.schulz@medizin.uni-leipzig.de) (AS)

**^¶^**These authors contributed equally to this work.

**Additional methods**

Immunohistochemistry of heart sections

Frozen tissue sections were postfixed in ice cold 4% neutral-buffered formalin for 5 min. Endogenous peroxidase activity was quenched for 30 min in a solution containing 3 % hydrogen peroxide and 10 % methanol. Nonspecific binding of reactive sites was prevented by PBS containing 1.5 % normal goat serum. Primary rat anti-murine CD68 antibody (10 µg/ ml, Bio-Rad, München, Germany) was applied overnight in a moist chamber at 4 °C. Primary antibody binding was located with a biotinylated goat anti-rat antibody (1:200, 30 min at room temperature, VEC-BA-4001; Biozol, Eching, Germany). Secondary antibody binding was visualized with avidin-biotin-horse radish peroxidase complex (Vectastain ABC kit; Biozol, see above) and diaminobenzidine solution. Cell nuclei were counterstained by Mayer's hemalaun before mounting in Roti®-Histokitt (Roth, Karlsruhe, Germany). The immunostained sections were evaluated under an Axioplan 2 microscope (Zeiss, Jena, Germany), equipped with a ProgRes C3 digital camera (Jenoptik, Jena, Germany) and connected to a digital recording system (ProgRes CapturePro 2.8.8, Jemoptik, as above).

RNA preparation and qPCR of aortae

Frozen aortic tissue (-80 °C) was placed in special tubes with 500 µl [TRI Reagent(R)](http://www.sigmaaldrich.com/catalog/product/sigma/t9424?lang=en&region=US) (Sigma-Aldrich, Germany) and ceramics beads. Tissue was disrupted with an Precellys 24 homogenizer (Peqlab, Erlangen, Germany) for two cycles of 90 seconds. Total RNA was prepared with the RNeasy Mini Kit (Qiagen, Hilden, Germany) according to the manual. One µg RNA was reverse transcribed in 40µl total volume using the Omniscript RT Kit (Qiagen). Quantitative PCR was performed with the following gene specific primers: for MCP1 (sense, 5’-AGCACCAGCCAACTCTCACT-3’; antisense 5’-TCATTGGGATCATCTTGCTG-3’) and TNF-α (sense, 5’-GAACTGGCAGAAGAGGCACT-3’; antisense 5’-GAGGCCATTTGGGAACTTCT-3’) on a BioRad CFX Connect instrument and data were analyzed with the CFX Manager software.
