# Supplementary material for: Severe Atherosclerosis and Hypercholesterolemia in Mice Lacking Both the Melanocortin Type 4 Receptor and Low Density Lipoprotein Receptor
Source: PLoS One. 2016 Dec 28;11(12):e0167888. doi: 10.1371/journal.pone.0167888 (PMC5193345; doi:10.1371/journal.pone.0167888)
Supplement: S1 Table — (DOCX) [file pone.0167888.s002.docx]

**S1 Table. Composition of the diets used.**

Regular chow and semisynthetic cholesterol-containing diet was purchased from Ssniff GmbH (Soest, Germany). Ingredients for each diet are listed in the table. # Metabolizable energy calculated according to the pig formula (Annex 4 of the German feed regulation)

| **Component** | **Regular chow diet R/M H** | **Semisynthetic diet (AIN76A)** |
| --- | --- | --- |
| **Gross energy** | **16.3 MJ/kg** | **19.0 MJ/kg** |
| **Metabolizable energy** | **12.8 MJ/kg#** | **16.2 MJ/kg#** |
| **Crude nutrients in %** | | |
| Dry matter | 87.7 | 96.8 |
| Crude protein | 19.0 | 17.4 |
| Crude fat | 3.3 | 5.1 |
| Crude fibre | 4.9 | 5.0 |
| Crude ash | 6.4 | 2.0 |
| N free extracts | 54.1 | 67.4 |
| Starch | 13.9 | 13.9 |
| Sugar | 4.7 | 51.0 |
| **Cholesterol** | **0%** | **0.02%** |
| **Minerals in %** | | |
| Calcium | 1.0 | 0.63 |
| Phosphorus | 0.7 | 0.55 |
| Sodium | 0.24 | 0.11 |
| Magnesium | 0.22 | 0.05 |
| Potassium | 0.91 | 0.36 |
| Ca:P |  | 1.14 |
| **Fattyacids in %** | | |
| C 14:0 | 0.01 | 0.01 |
| C 16:0 | 0.47 | 0.57 |
| C 16:1 | 0.01 | 0.01 |
| C 18:0 | 0.08 | 0.10 |
| C 18:1 | 0.62 | 1.31 |
| C 18:2 | 1.80 | 2.77 |
| C 18:3 | 0.23 | 0.05 |
| C 20:0 | 0.01 | 0.02 |
| C 20:1 | 0.02 | - |
| **Aminoacids in %** | | |
| Lysine | 1.00 | 1.43 |
| Methionine | 0.30 | 0.82 |
| Cystine | not specified | 0.08 |
| Met+Cys | 0.65 | 0.90 |
| Threonine | 0.68 | 0.77 |
| Tryptophan | 0.25 | 0.22 |
| Agrinine | 1.14 | 0.63 |
| Histidine | 0.44 | 0.55 |
| Valine | 0.88 | 1.18 |
| Isoleucine | 0.76 | 0.90 |
| Leucine | 1.30 | 1.70 |
| Phenylalanine | 0.85 | 0.93 |
| Phe+Tyr | 1.43 | 1.85 |
| Glycine | 0.80 | 0.36 |
| Glutamicacid | 3.90 | 3.90 |
| Asparticacid | 1.61 | 1.29 |
| Proline | 1.25 | 2.00 |
| Alanine | 0.79 | 0.57 |
| Serine | 0.89 | 1.03 |
| **Vitamins per kg** | | |
| Vitamin A | 15,000 IU | 4,000 IU |
| Vitamin D3 | 1,000 IU | 1,000 IU |
| Vitamin E | 110 mg | 57 mg |
| Vitamin K (asmenadione) | 5 mg | 5 mg |
| Thiamin (B1) | 18 mg | 3 mg |
| Riboflavin (B2) | 23 mg | 5 mg |
| Pyridoxine (B6) | 21 mg | 6 mg |
| Cobalamin (B12) | 100 µg | 10 µg |
| Nicotinicacid | 135 mg | 29 mg |
| Pantothenicacid | 43 mg | 15 mg |
| Folicacid | 7 mg | 2 mg |
| Biotin | 525 µg | 20 µg |
| Choline-Chloride | 2,990 mg | 1,040 mg |
| Inositol | 100 mg | not specified |
| **Trace elements per kg** | | |
| Iron | 179 mg | 46 mg |
| Manganese | 69 mg | 59 mg |
| Zinc | 94 mg | 35 mg |
| Copper | 16 mg | 7 mg |
| Iodine | 2.2 mg | 0.22 mg |
| Selenium | 0.3 mg | 0.14 mg |
| Cobalt | 2.1 mg | 0.02 mg |
