# Supplementary material for: Severe Atherosclerosis and Hypercholesterolemia in Mice Lacking Both the Melanocortin Type 4 Receptor and Low Density Lipoprotein Receptor
Source: PLoS One. 2016 Dec 28;11(12):e0167888. doi: 10.1371/journal.pone.0167888 (PMC5193345; doi:10.1371/journal.pone.0167888)
Supplement: S2 Table — (DOCX) [file pone.0167888.s003.docx]

**S2 Table. ANOVA analysis of factors influencing body weight and length.**

To analyze whether the weight gain and body length differences are statistically different and dependent of factors like sex, genotypes and diet we used a 3-way ANOVA into which we included the interaction between the three factors. Visual inspection of qq-plots and residuals plotted against fitted values revealed no obvious deviations from the assumptions of normally distributed and homogeneous residuals. Prior to inspecting the significance of the individual terms we established their significance as a whole by comparing the full model with the null model comprising only the intercept. The analysis was run in R (version 3.1.0, [[32](#_ENREF_32)]). The overall comparison between the full and the null model was highly significant for weight (F _15,182_ = 40.60, p = 2.2e-16) and for length (F _15, 182_ = 13.42, p = 2.2e-16). Df = degrees of freedom

|  | **Body weight** | | | | **Body length** | | | | | |
| --- | --- | --- | --- | --- | --- | --- | --- | --- | --- | --- |
|  | Df | F value | p-value | | Df | F value | | p-value | |  |
| sex | 1 | 94.5 | <2.2e-16 | *** | 1 | 38.9 | 3.6E-09 | | *** | |
| diet | 1 | 18.8 | 2.4E-05 | *** | 1 | 6.3 | 1.3E-02 | | * | |
| genotype | 3 | 152.6 | <2.2e-16 | *** | 3 | 43.8 | <2.2e-16 | | *** | |
| sex:diet | 1 | 4.3 | 4.0E-02 | * | 1 | 4.2 | 4.3E-02 | | * | |
| sex:genotype | 3 | 5.7 | 1.0E-03 | *** | 3 | 4.0 | 8.4E-03 | | ** | |
| diet:genotype | 3 | 4.1 | 7.8E-03 | ** | 3 | 0.8 | 4.7E-01 | |  | |
