# Supplementary material for: Severe Atherosclerosis and Hypercholesterolemia in Mice Lacking Both the Melanocortin Type 4 Receptor and Low Density Lipoprotein Receptor
Source: PLoS One. 2016 Dec 28;11(12):e0167888. doi: 10.1371/journal.pone.0167888 (PMC5193345; doi:10.1371/journal.pone.0167888)
Supplement: S3 Table — (DOCX) [file pone.0167888.s004.docx]

**S3 Table. Results of the multiple regression analysis for plaque size measured in BCA and heart.**

For multiple regression analysis plaque size was compared with different other parameters for heart and BCA (cholesterol, diet, sex, genotype). Plaque size was positively influenced by cholesterol (elevation of serum cholesterol by 1 mM). Diet changes from semisynthetic diet to chow, gender comparison from females to males and *Mc4r^mut^;Ldlr^-/-^* to *Ldlr^-/-^* reduced plaque size as shown by negative values. Exact p-values for each comparison are given in the Pr(>|t|) column. Genotype differences were not significant. * p < 0.05** p < 0.01*** p < 0.001

| **BCA** | **Estimate** | **Std.Error** | **t.value** | **Pr(>\|t\|)** |  |
| --- | --- | --- | --- | --- | --- |
| (Intercept) | 7991.6 | 3937.7 | 2.029 | 4.5E-02 | * |
| serum cholesterol | 759.6 | 120.6 | 6.296 | 9.7E-09 | *** |
| diet (semi - chow) | -8156.3 | 2960.3 | -2.755 | 7.0E-03 | ** |
| sex (female - male) | -8055.5 | 2222.2 | -3.625 | 4.7E-04 | *** |
| genotype (*Mc4r^mut^*;*Ldlr^-/-^* - *Ldlr^-/-^)* | -209.7 | 2471.2 | -0.085 | 9.3E-01 |  |
|  |  |  |  |  |  |
| **Heart** | **Estimate** | **Std.Error** | **t.value** | **Pr(>\|t\|)** |  |
| (Intercept) | 222697 | 36520 | 6.098 | 3.5E-08 | *** |
| serum cholesterol | 6682 | 1083 | 6.169 | 2.6E-08 | *** |
| diet (chow) | -130841 | 27412 | -4.773 | 7.9E-06 | *** |
| sex (male) | -143434 | 20678 | -6.937 | 8.9E-10 | *** |
| genotype (*Ldlr^-/-^*) | -35988 | 22914 | -1.571 | 1.2E-01 |  |
