# Supplementary material for: Severe Atherosclerosis and Hypercholesterolemia in Mice Lacking Both the Melanocortin Type 4 Receptor and Low Density Lipoprotein Receptor
Source: PLoS One. 2016 Dec 28;11(12):e0167888. doi: 10.1371/journal.pone.0167888 (PMC5193345; doi:10.1371/journal.pone.0167888)
Supplement: S4 Table — (DOCX) [file pone.0167888.s005.docx]

**S4 Table. Results of spearman correlation between serum cholesterol levels and the plaque size in BCA and heart, respectively.**

Animal numbers, correlation coefficients and p-value of the analysis are summarized in this table and illustrated in Fig 4.

|  |  |  | **female** | |  | **male** | |
| --- | --- | --- | --- | --- | --- | --- | --- |
|  |  |  | ***Mc4r^mut^; LDL^-/-^*** | ***LDL^-/-^*** |  | ***Mc4r^mut^; LDL^-/-^*** | ***LDL^-/-^*** |
| **Heart** | **n** |  | 23 | 17 |  | 19 | 27 |
|  | **r^2^** |  | 0.82 | 0.79 |  | 0.5 | 0.76 |
|  | **p-value** |  | 3.76E-09 | 1.69E-06 |  | 7.19E-04 | 3.15E-09 |
|  |  |  |  |  |  |  |  |
| **BCA** | **n** |  | 26 | 22 |  | 24 | 28 |
|  | **r^2^** |  | 0.53 | 0.37 |  | 0.73 | 0.67 |
|  | **p-value** |  | 2.58E-03 | 2.85E-03 |  | 1.17E-07 | 1.00E-07 |
