# Supplementary material for: Severe Atherosclerosis and Hypercholesterolemia in Mice Lacking Both the Melanocortin Type 4 Receptor and Low Density Lipoprotein Receptor
Source: PLoS One. 2016 Dec 28;11(12):e0167888. doi: 10.1371/journal.pone.0167888 (PMC5193345; doi:10.1371/journal.pone.0167888)

**S1 Fig. Body weight development.**

Body weight development was measured in all groups from weaning up to an age of 6 months. The upper graph shows body weight curves for males, the lower graph for females under regular chow and semisynthetic diet (cholesterol-containing). Data are presented as mean ± SEM for each group (n = 10 -15 animals).


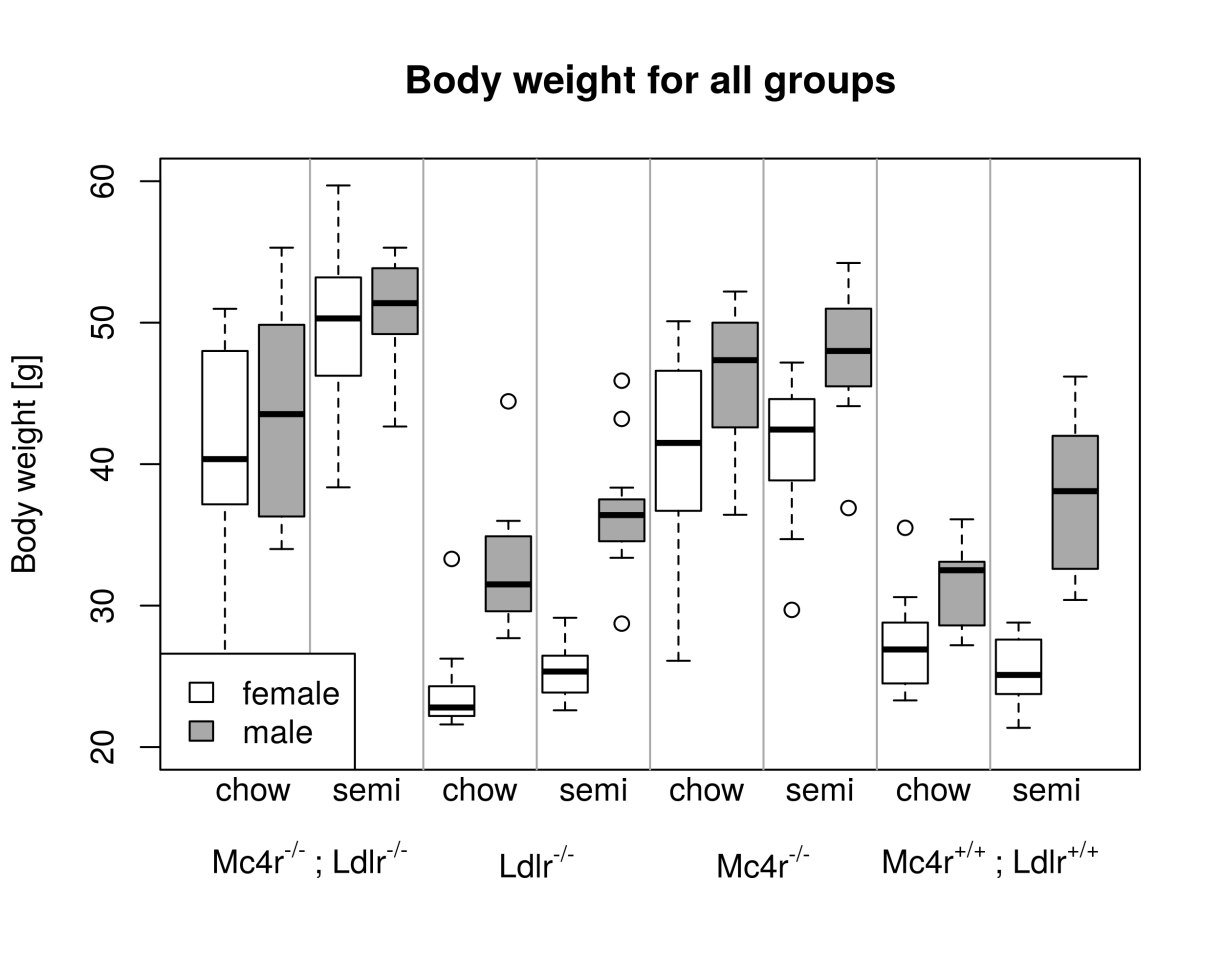

Supplement: S1 Fig — (DOCX) [file pone.0167888.s006.docx]
