# Supplementary material for: Severe Atherosclerosis and Hypercholesterolemia in Mice Lacking Both the Melanocortin Type 4 Receptor and Low Density Lipoprotein Receptor
Source: PLoS One. 2016 Dec 28;11(12):e0167888. doi: 10.1371/journal.pone.0167888 (PMC5193345; doi:10.1371/journal.pone.0167888)
Supplement: S3 Fig — (DOCX) [file pone.0167888.s008.docx]

**
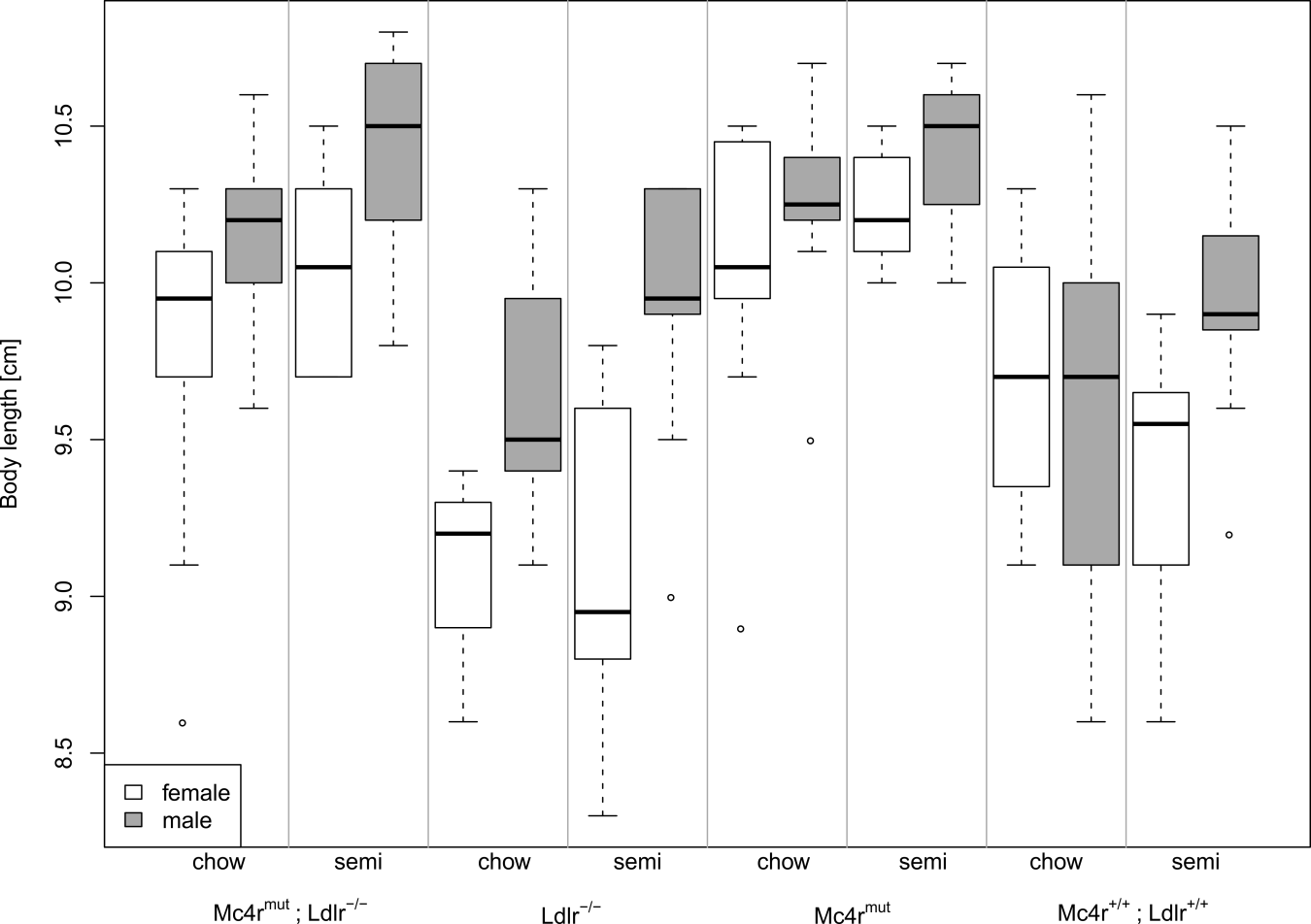
**

**S3 Fig. Body length of all groups.**

In the figure Box-Whisker-Plots for body length for all groups and both diets are shown. Center lines show the medians; box limits indicate the 25th and 75th percentiles and whiskers extend to minimum and maximum values up to 1.5 times the interquartile range above the upper quartile and below the lower quartile. More extreme values are shown as outliers.
