# Supplementary material for: Severe Atherosclerosis and Hypercholesterolemia in Mice Lacking Both the Melanocortin Type 4 Receptor and Low Density Lipoprotein Receptor
Source: PLoS One. 2016 Dec 28;11(12):e0167888. doi: 10.1371/journal.pone.0167888 (PMC5193345; doi:10.1371/journal.pone.0167888)
Supplement: S4 Fig — (DOCX) [file pone.0167888.s009.docx]

| A | B |
| --- | --- |
| 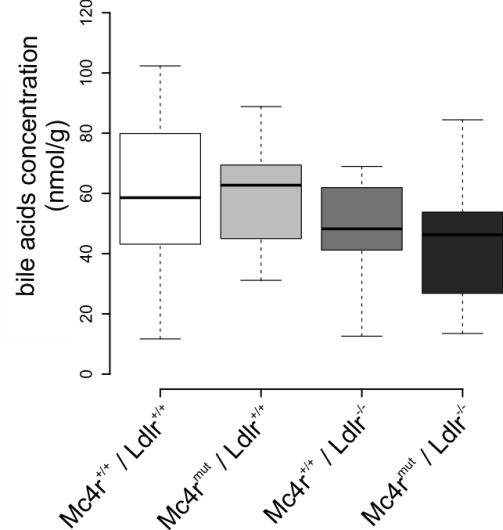 |  |

**S4 Fig. Measurement of bile acids.**

Bile acids were determined from liver tissue and gall bladder of chow feeded mice. A) Measurement of bile acids in liver tissue. Weighted pieces of frozen liver tissue were homogenized in PBS, centrifuged and bile acids concentration was measured in the supernatant.

B) Bile was collected from scarified mice by puncture of the gall bladder with a syringe and vials were stored at -80°C until use. Bile acids measurements were performed with an enzymatic kit from Diazyme Europe GmbH (Dresden, Germany) according to the manufacturer’s instructions.
