# Supplementary material for: Severe Atherosclerosis and Hypercholesterolemia in Mice Lacking Both the Melanocortin Type 4 Receptor and Low Density Lipoprotein Receptor
Source: PLoS One. 2016 Dec 28;11(12):e0167888. doi: 10.1371/journal.pone.0167888 (PMC5193345; doi:10.1371/journal.pone.0167888)
Supplement: S5 Fig — (DOCX) [file pone.0167888.s010.docx]

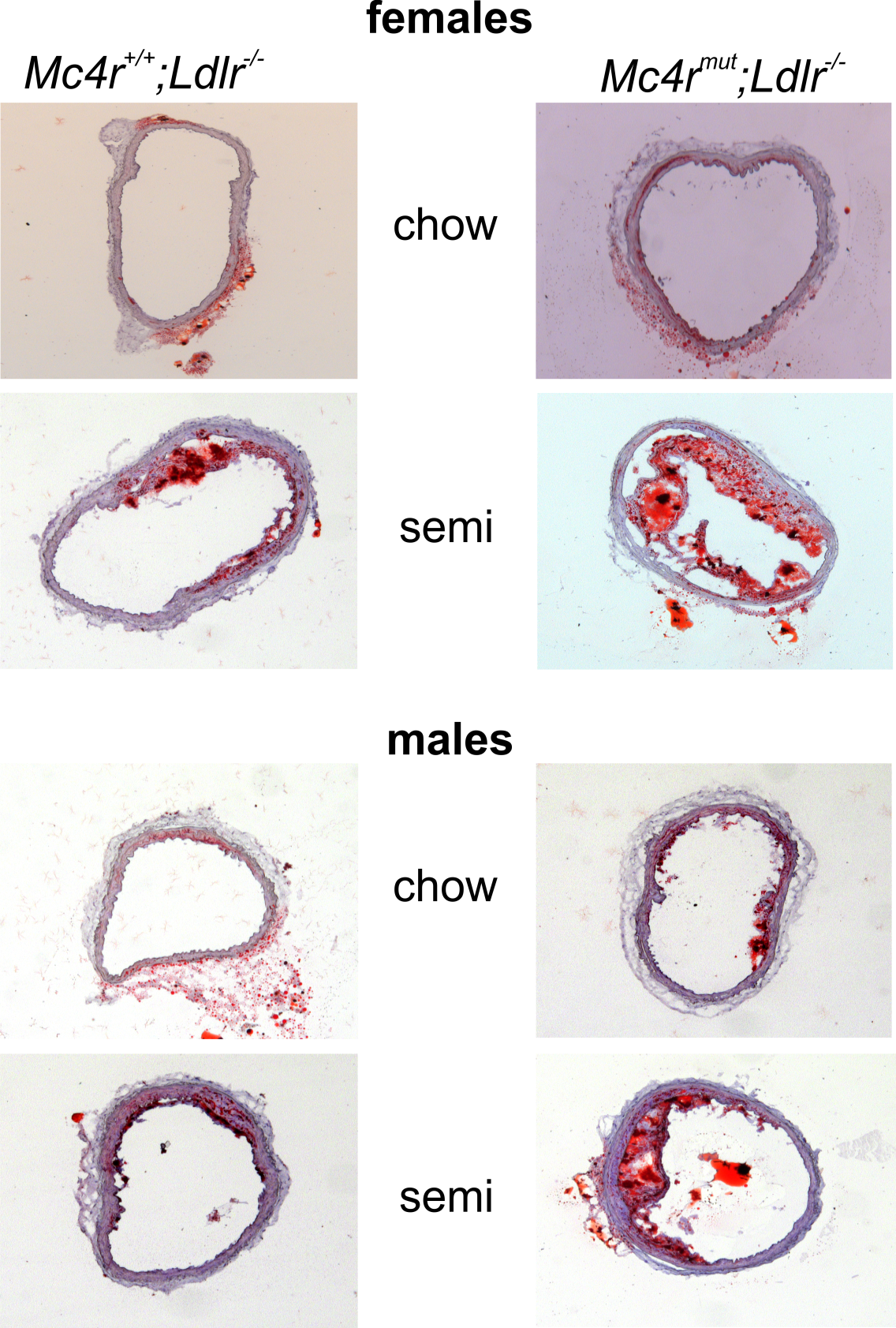


### S5 Fig. Atherosclerosis in mice lacking both receptors Mc4r and Ldlr.

Representative sections of the brachiocephalicartery (BCA) prepared from the different mouse groups. Depicted are BCA of *Ldlr*^-/-^ and *Mc4r^mut^*;*Ldlr*^-/-^ mice of both genders fed a chow and semisynthetic diet (0.02% cholesterol).
