# Supplementary material for: Severe Atherosclerosis and Hypercholesterolemia in Mice Lacking Both the Melanocortin Type 4 Receptor and Low Density Lipoprotein Receptor
Source: PLoS One. 2016 Dec 28;11(12):e0167888. doi: 10.1371/journal.pone.0167888 (PMC5193345; doi:10.1371/journal.pone.0167888)
Supplement: S6 Fig — (DOCX) [file pone.0167888.s011.docx]

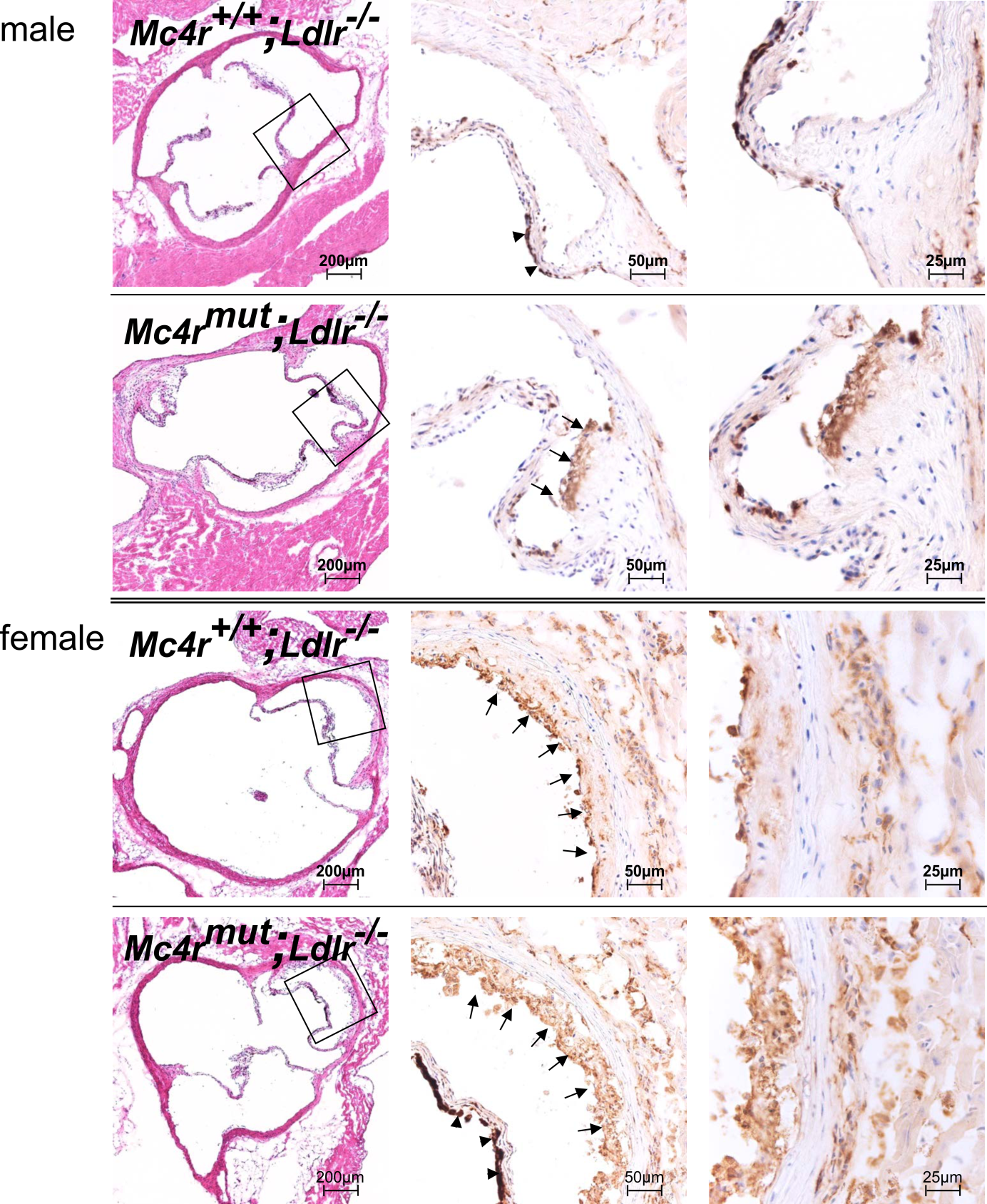


### S6 Fig. Immunohistochemistry of hearts of Mc4r wildtype and mutant mice.

Detection of CD68 expression in atherosclerotic aortic root lesions of male and female mice expressing wild type (row 1 and 3) and mutant *Mc4r* (row 2 and 4) on *Ldlr^-/-^* background under chow diet, respectively. In each row the HE stained section on the left provides overview information about the CD68 stained area depicted (rectangle) in the sections next to it. In both genders, the plaque compositions in terms of CD68 (arrows) is more pronounced in mutant *Mc4r* mice compared to wild type littermates. The arrowheads point to melanin-producing cells frequently identified in the subendothelial layer of C57/BL6 aortic valves cups. All sections in the first column are stained with H&E, sections in the second and third row are immunostained with anti-CD68 antibody and counterstained by Mayer's hemalaun. Scale bars as indicated.
