# Supplementary material for: Severe Atherosclerosis and Hypercholesterolemia in Mice Lacking Both the Melanocortin Type 4 Receptor and Low Density Lipoprotein Receptor
Source: PLoS One. 2016 Dec 28;11(12):e0167888. doi: 10.1371/journal.pone.0167888 (PMC5193345; doi:10.1371/journal.pone.0167888)
Supplement: S7 Fig — (DOCX) [file pone.0167888.s012.docx]

### S7 Fig. Gene expression data from aortae of all genotypes.

Total RNA from aortae of all genotypes was prepared as stated in *Additional methods*. Quantitative PCR reactions were performed for MCP1 and TNF-α with exon-specific primers. Eight (*Mc4r^+/+^* and *Mc4r^mut^)* or nine samples (*Mc4r^+/+^*;*Ldlr^-/-^* and *Mc4r^mut^*;*Ldlr^-/-^)* per group and gender were analyzed. In cases were the specific gene could not be detected in all samples of the group, number of samples in relation to the total group number is given below the column. Shown are delta Ct values in relation to ß2 microglobulin, used as housekeeping gene. nd – not detectable
